# DNA Targeting Sequence Improves Magnetic Nanoparticle-Based Plasmid DNA Transfection Efficiency in Model Neurons

**DOI:** 10.3390/ijms160819369

**Published:** 2015-08-17

**Authors:** Matthew M. Vernon, David A. Dean, Jon Dobson

**Affiliations:** 1 J. Crayton Pruitt Family Department of Biomedical Engineering, University of Florida, Gainesville, FL 32611, USA; E-Mail: vernonmm@gmail.com; 2 School of Medicine and Dentistry, University of Rochester Medical Center, Rochester, NY 14642, USA; E-Mail: david_dean@urmc.rochester.edu; 3 Department of Materials Science and Engineering, University of Florida, Gainesville, FL 32603, USA; 4 Institute for Cell Engineering & Regenerative Medicine (ICERM), University of Florida, Gainesville, FL 32611, USA

**Keywords:** DNA targeting sequence, gene transfection, magnetic nanoparticles, neuroblastoma, nuclear localization, primary neurons, plasmid DNA, SH-SY5Y cells

## Abstract

Efficient non-viral plasmid DNA transfection of most stem cells, progenitor cells and primary cell lines currently presents an obstacle for many applications within gene therapy research. From a standpoint of efficiency and cell viability, magnetic nanoparticle-based DNA transfection is a promising gene vectoring technique because it has demonstrated rapid and improved transfection outcomes when compared to alternative non-viral methods. Recently, our research group introduced oscillating magnet arrays that resulted in further improvements to this novel plasmid DNA (pDNA) vectoring technology. Continued improvements to nanomagnetic transfection techniques have focused primarily on magnetic nanoparticle (MNP) functionalization and transfection parameter optimization: cell confluence, growth media, serum starvation, magnet oscillation parameters, *etc.* Noting that none of these parameters can assist in the nuclear translocation of delivered pDNA following MNP-pDNA complex dissociation in the cell’s cytoplasm, inclusion of a cassette feature for pDNA nuclear translocation is theoretically justified. In this study incorporation of a DNA targeting sequence (DTS) feature in the transfecting plasmid improved transfection efficiency in model neurons, presumably from increased nuclear translocation. This observation became most apparent when comparing the response of the dividing SH-SY5Y precursor cell to the non-dividing and differentiated SH-SY5Y neuroblastoma cells.

## 1. Introduction

Gene therapy involves alteration of the amount or type of protein expression in a target cell population for the ultimate purpose of disease treatment. Unfortunately, the challenge of obtaining high transfection rates in embryonic stem cells, mesenchymal stem cells, primary/progenitor neurons and the majority of primary cells hinders research progress in the area of gene therapy [1,2]. The SH-SY5Y cell line has modeled the neuron in many research applications where the cost or difficultly of culturing primary neurons are limiting factors. Accordingly, the optimization parameters uncovered in this study would likely also improve transfection outcomes for primary neurons and potentially other post-mitotic cell phenotypes that are challenging to transfect [3].

Oscillating array-based magnetic nanoparticle DNA transfection is a gene vectoring technique that is promising because it is capable of outperforming most other non-viral transfection methods, in terms of both transfection efficiency and cell viability [4]. Nanomagnetic transfection starts with the electrostatic association of plasmid DNA (pDNA) and magnetic nanoparticles (MNPs) in aqueous suspension to form pDNA-MNP complexes. Next, these complexes are added to culture plates containing the *in vitro* target cell population. Then, the addition of a magnet array below the cell culture plate results in sedimentation of the pDNA-MNP complexes, thereby forcing sustained and proximal contact of the transgene vector and target cell [5,6,7]. Finally, one-dimensional oscillation of the magnet array produces movement of the pDNA-MNP complexes at the targeted cells’ surface that facilitates endocytosis via mechanical stimulation [4,8].

Improvements to this technique have occurred mostly with transfection parameter optimization and magnetic particle functionalization, which cannot assist in the nuclear translocation of the naked pDNA from the cytoplasm [9]. In post-mitotic cells, researchers have observed that successful plasmid translocation and infiltration into the nucleus becomes a significant barrier to transfection [10,11,12]. Accordingly, inclusion of a nuclear localization sequence of specific nucleotides within the delivered transgenic plasmid DNA may potentially improve transfection efficiency in post-mitotic cell phenotypes [13].

**Figure 1:**
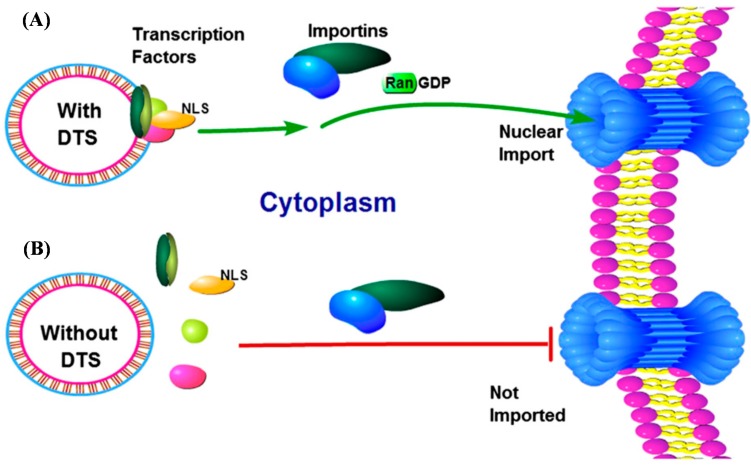
Schematic representation of plasmid DNA nuclear import. (**A**) Incorporation of specific promoter fragments, known as a DTS feature, contain binding sites for transcription factors expressed in target cells. Following cytoplasmic delivery of the transgenic plasmid DNA, native transcription factors from the host-cell bind to the plasmid DNA and present NLSs. Next, endogenous importins form a protein complex that recognize the NLSs and shuttle the plasmid into the host-cell nucleus via the nuclear pore complex (NPC); (**B**) Conventional plasmids without a DTS feature cannot form the protein-pDNA complex required for importin-mediated nuclear translocation [15].

Researchers have already identified many nuclear localization signals (NLS) that mammalian cells use to identify materials tagged for nuclear import. The simian vacuolating virus 40 (SV40) origin of replication region containing the enhancer and the early and late promoter sequences was the first DNA based nuclear targeting signal identified and isolated [14]. When incorporated into cytoplasmically delivered transgenic plasmids, the 72 bp SV40 enhancer alone confers nuclear localization to the delivered plasmid in all cell types tested. The SV40-dervied DNA targeting sequence (DTS) consists of consensus binding sites for numerous transcription factors found universally in mammalian cells. Specifically, the SV40DTS can interact with AP1, AP2, AP3, AP4, NF-κB, Oct-1 and SP1 transcriptional factors. As shown in Figure 1, the DTS feature of the delivered plasmid recruits native transcription factors that bind and present NLSs. Then, specific importin proteins form a complex that interacts with the newly formed NLSs and Ran-GDP to shuttle the plasmid from the cytoplasm into the nucleus via the nuclear pore complex (NPC) [15].

Later, the identification of other DTSs led to the discovery that many only demonstrate activity in specific cells [13]. For example, the mechanism for nuclear translocation of the SMGADTS plasmid relies upon the binding of two smooth muscle cell transcription factors (SRF and Nkx3.1/3.2) to a region of the smooth muscle gamma actin (SMGA) promoter. This leads to importin-mediated nuclear translocation of the cytoplasmic plasmid in smooth muscle cells, but does not occur in cell types that lack these two transcription factors [16]. This *in vitro* phenomenon was confirmed through an *in vivo* transfection of rat vasculature. Specifically, SMGADTS plasmid expression was found only in smooth muscle cells, despite the presence of many other cell types *in vivo* that received the SMGADTS construct [17]. Therefore, a cell-specific DTS allows for an additional control mechanism over precise transfection of a cell subpopulation *in vivo*. Accordingly, three plasmids will be tested in the present study: noDTS, SV40DTS and SMGADTS. The noDTS plasmid lacks a DTS feature, which provides a suitable negative control to the DTS-containing plasmid SV40DTS [18]. The SMGADTS plasmid also serves as a negative control, because the DTS feature within the construct only displays activity in smooth muscle cells that express gamma actin. Consequently, the DTS of the SMGA plasmid is not expected to display activity in the target neuroblastoma cells, since they do not express the gamma actin microfilament [16]. Finally, the SV40DTS contains a non-specific DTS that has demonstrated enhanced transfection via importin-mediated nuclear translocation in a variety of tested cell types [19].

Administration of all-*trans* retinoic acid (ATRA) followed sequentially by brain-derived neurotrophic factor (BDNF) to the SH-Y5Y cell culture induces differentiation. The neuroblastoma cells undergo many changes during this differentiation procedure to emerge with the physical and behavioral properties of specialized neuronal cells [20,21]. In post-mitotic differentiated SH-SY5Y cells, the nuclear membrane presents a primary barrier for transgene delivery to the nucleus. Therefore, incorporation of a DNA targeting sequence within the plasmid DNA may potentially lead to improved transfection outcomes through nuclear translocation via the importin pathway [13]. The SH-SY5Y cell line is ideally suited for testing this hypothesis, as the intrinsic relationship of the undifferentiated and differentiated SH-SY5Y cell phenotypes allows for a controlled experimental comparison for modeling both a dividing and a post-mitotic neural phenotype.

In the present study, three nanoparticle vectors are tested for their transfection efficiency of undifferentiated and differentiated neuroblastoma cells. The commercially available nanoparticle vectors: Polymag Neo, nTMag and Neuromag have varying physical and electrostatic properties that likely account for the observed differences in performance. In addition to exploring the transfection efficiency of these three nanoparticle vectors, the effect of including a DTS sequence within the transfecting plasmid is also tested. The selection of a nanoparticle vector can affect transfection outcome, because the interaction of the vector with the cell membrane determines the efficiency of internalization and endosomal escape [22]. Once the transfecting nanoparticle and plasmid disassociate in the cytoplasm following endosomal escape, the inclusion of a DTS sequence facilitates nuclear translocation to ultimately improve the transfection outcome [13]. Therefore, the aims of this study are to compare different MNPs in their ability to transfect neuroblastoma cells and assess the potential benefit of incorporating a DTS signal in the delivered plasmid.

## 2. Results and Discussion

### 2.1. Transgenic Plasmid Size Primarily Determines Nanomagnetic Transfection Outcome in the Undifferentiated SH-SY5Y Cell Phenotype

**Figure 2:**
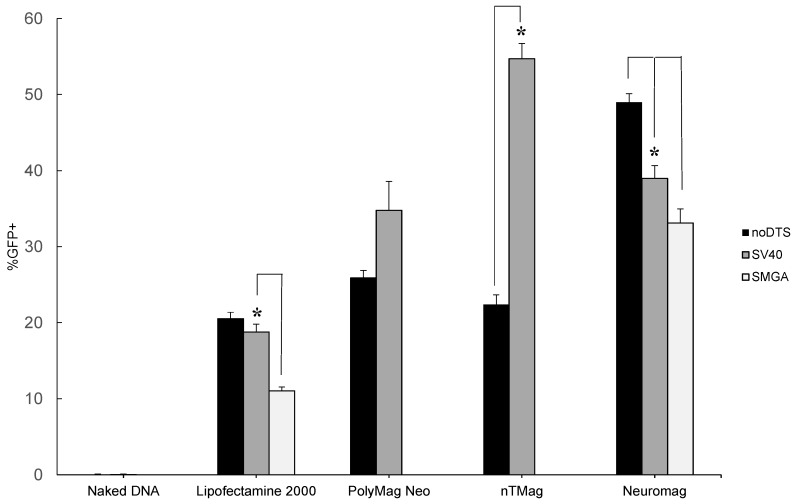
Summary of flow cytometry data for undifferentiated SH-SY5Y cell transfection efficiency assayed at days *in vitro* (DIV) 2 post-transfection. Nanomagnetic plasmid delivery was conducted with the Magnefect-nano II™ controller unit with the transfection parameters of 2 Hz frequency, 0.2 mm displacement and a duration of 3600 cycles or 30 min. All vector and plasmid combinations were investigated for transfection efficiency, except for the SMGADTS plasmid that was not tested with the PolyMag Neo and nTmag nanoparticle vectors. Error expressed as standard deviation from the mean, where *n* = 3 for all samples. Statistical significance determined using 2-sample *t*-test, with a confidence interval of * *p* ≤ 0.05.

The undifferentiated SH-SY5Y cell readily divides *in vitro*, which conveniently allows researchers to propagate the cell line to meet experimental demand. Transfection of undifferentiated SH-SY5Y cells produced mixed results, as gene delivery with DTS-containing plasmids does not consistently outperform a standard transgene reporter plasmid. Intriguingly, the performance of the control transgene reporter *vs.* a DTS-containing plasmid can vary dramatically dependent on the vector implemented. Specifically, in Figure 2 the noDTS, SV40 and SMGA plasmids demonstrated similar transgene expression when delivered with Lipofectamine™-2000, PolyMag Neo or Neuromag. These observations likely stem from the properties of the nanoparticle vectors (see Table 1). The PolyMag Neo and Neuromag vectors have similar size and zeta potential parameters, while the nTMag vector possess both a smaller size and less electrostatic charge. Nanoparticle vector size and zeta potential play an important role in the electrostatic association and disassociation of the nanoparticle vector to pDNA cargo as well as the host-cell membrane interactions that precede endocytosis [23,24,25]. These comparable vector attributes combined with the mitotic nature of the undifferentiated SH-SY5Y cell may explain the uniformity of plasmid performance for each of the tested vectors.

**Table 1:** Summary of measured magnetic nanoparticle vector properties.

MNP Type	Mode Diameter (nm)	Mean Diameter (nm)	Size Range (nm)	Zeta Potential (mV)
PolyMag Neo	146	166.21	105–225	+49.29
nTMag	99	119.41	70–170	+31.13
Neuromag	122	151.63	80–220	+48.16

The most plausible explanation for the observed variance among the tested plasmids is because of the size discrepancy. For example, the SMGADTS plasmid is the largest at 7002 bp, as compared to noDTS and SV40DTS that are similarly sized at 4609 and 5029 bp, respectively. Possibly, during vector and plasmid DNA conjugation more copies of the smaller noDTS and SV40 plasmids would associate with a given nanoparticle vector than compared to a larger plasmid with a similar sequence, such as SMGADTS. Therefore, it is conceivable that noDTS and SV40 nanomagnetic transfection outperform the SMGA plasmid because more plasmids are adsorbed electrostatically on the surface of the MNP vector during conjugation. Following endosomal escape of the MNP-pDNA into the cytoplasm, a greater amount of pDNA copies may disassociate from the nanoparticle carrier. Furthermore, plasmid diffusion rate within the cytoplasm is a size dependent process that might also contribute to this observation [26]. Ultimately among the performance of plasmids tested, activity of the SV40-derived DTS feature in the SV40DTS plasmid appeared to be overshadowed by the size dependent effects.

Lipofectamine™-2000 served as a positive control for this experiment, since use of this lipid-based transfection agent is common. In the present study, a transfection efficiency of 10%–20% was observed for Lipofectamine™-2000 (see Figure 2), which is comparable to the findings of other researchers who reported undifferentiated SH-SY5Y cell transfection efficiencies ranging from 0.3%–4% [27,28]. Furthermore, the superior performance of the nanoparticle vectors, in particular Neuromag, is revealed when compared to the established Lipofectamine™-2000 control (see Figure 2).

In contrast, reporter expression by the nTMag particle varied significantly depending on the plasmid DNA implemented. In Figure 2, the efficiency of transfection as measured via flow cytometric analysis increased from 22.4% ± 1.27% with the noDTS plasmid to 54.7% ± 2.02% for the SV40DTS plasmid. Since the undifferentiated SH-SY5Y cells are mitotic, the presence of a DTS signal in the delivered plasmid should not appreciably change the resulting gene expression. Potentially, the association during complex conjugation and/or dissociation kinetics of the SV40DTS plasmid with the nTMag nanoparticle account for this observed variance. Table 1 provides additional support for this theory, since nTMag has a smaller hydrodynamic size range and zeta potential magnitude, where both properties have implications in pDNA binding/unbinding kinetics and endocytosis rate [22,29].

### 2.2. Incorporation of a DTS Feature within the pDNA Improves Nanomagnetic Transfection Efficiency of the Model Neuron, Differentiated SH-SY5Y

**Figure 3:**
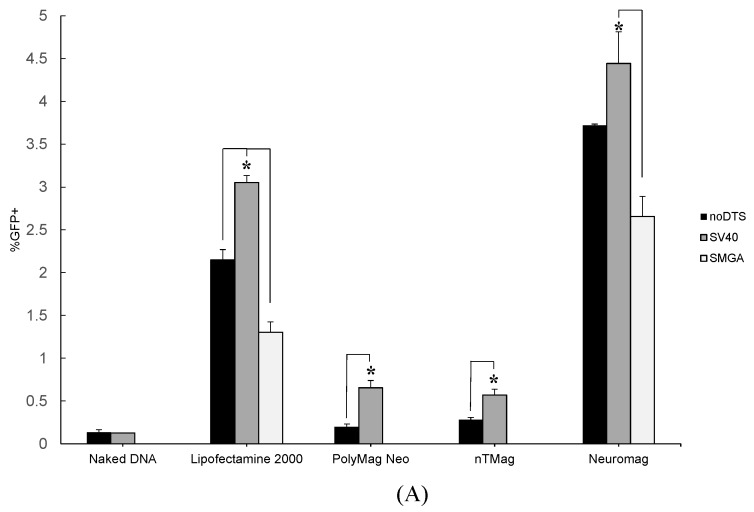

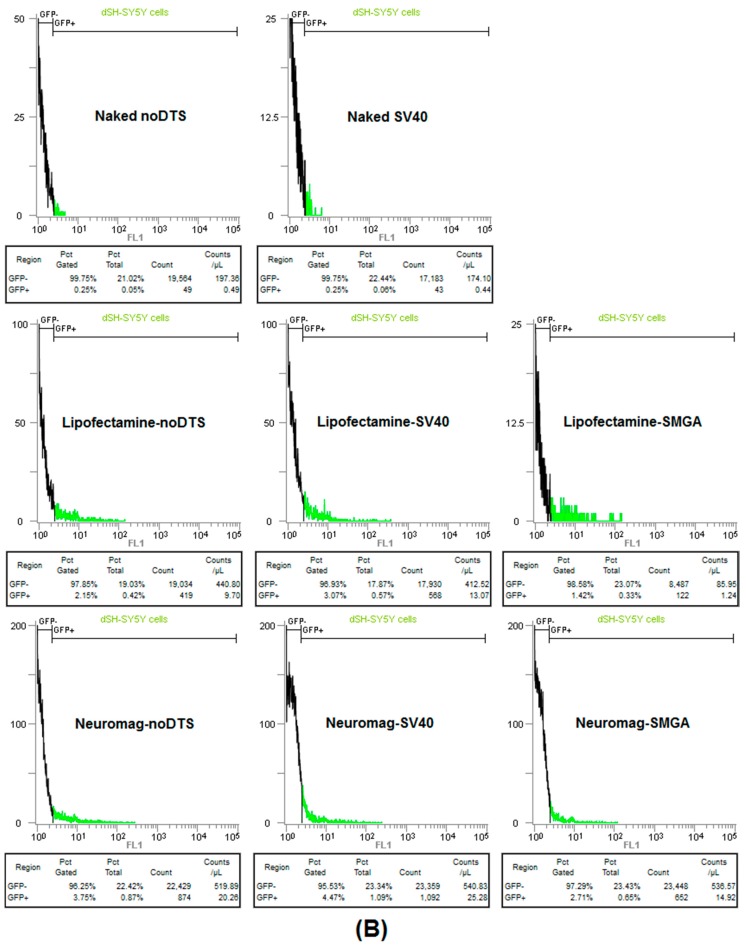

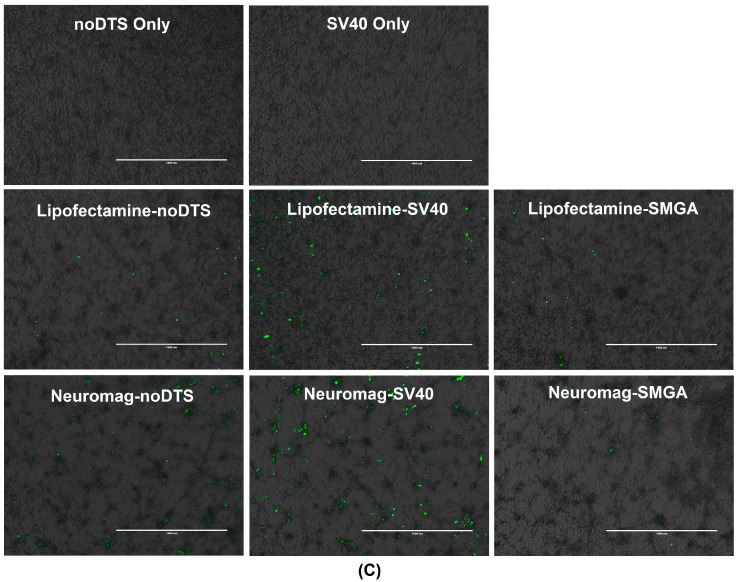
(**A**) Summary of flow cytometry data for differentiated SH-SY5Y cell transfection efficiency *vs.* vector type. Nanomagnetic plasmid delivery was conducted with the Magnefect-nano II™ controller unit with the transfection parameters of 2 Hz frequency, 0.2 mm displacement and a duration of 3600 cycles or 30 min. All vector and plasmid combinations were investigated for transfection efficiency, except for the SMGADTS plasmid that was not tested with the PolyMag Neo and nTmag nanoparticle vectors. Error expressed as standard deviation from the mean, where all data analysis was performed at DIV 6 post-transfection and *n* = 3 for all sample means. Statistical significance determined using two-sample *t*-test, with a confidence interval of * *p* ≤ 0.05; (**B**) Representative flow cytometry data plots of measured green fluorescent protein (GFP) signal resulting from naked DNA, Lipofectamine™-2000 and Neuromag transfection of differentiated neuroblastoma cells; (**C**) Representative differential interference contrast (DIC) and fluorescent microscopy overlay images from naked DNA, Lipofectamine™-2000 and Neuromag transfection of differentiated neuroblastoma cells. Scale bars: 1000 µm.

Unlike the rapidly-dividing undifferentiated neuroblastoma precursor, the differentiated SH-SY5Y cell phenotype does not readily proliferate. The post-mitotic state of the differentiated SH-SY5Y cell phenotype results in the constant presence of a nuclear membrane that functions as a significant barrier to transfection [10,11,12]. Accordingly, there was reduced overall efficiency of the noDTS, SV40DTS and SMGADTS reporter plasmids when vectored into model primary neurons. However, individual transgenic pDNAs displayed more consistent transfection performance across vector types tested on the differentiated neuroblastoma. Flow cytometric analysis (Figure 3A) and fluorescent imaging (Figure 3C) reveal enhanced transfection with the SV40DTS plasmid *vs.* control plasmids for all vectors. This differs from the previous observation shown in Figure 2, where the noDTS plasmid marginally outperformed SV40DTS across vector types when transfecting undifferentiated SH-SY5Y cells. A possible explanation arises when noting the smaller size of the noDTS plasmid (4609 bp), compared to SV40DTS (5029 bp) and SMGADTS (7002 bp), which might allow for more electrostatic adherence opportunities for pDNA molecules per nanoparticle, greater plasmid unloading and increased cytoplasmic diffusional velocity [26]. Adsorption of more plasmid copies per nanoparticle vector may result in the release of more plasmid copies into the cytoplasm when transfecting undifferentiated SH-SY5Y cells. This may ultimately manifest in higher transfection efficiency with the smaller noDTS than compared to the SV40DTS and SMGADTS plasmids. However, as shown in Figure 3A the different association/disassociation kinetics of individual plasmids with nanoparticle vectors appears to be overshadowed by the effect of a DTS feature in the transfecting plasmid for differentiated neuroblastoma cells.

The enhancement of transfection efficiency by the DTS-containing plasmid over the noDTS and SMGADTS control plasmids is readily apparent for all nanoparticle vectors tested when transfecting differentiated neuroblastoma cells (see Figure 3A). The SV40DTS plasmid outperformed the control plasmids in all trials except for the noDTS-SV40 comparison with the Neuromag vector, which still follows the same trend, as confirmed by applying a 2-sample *t*-test that generated a *p*-value of 0.077. Since a functional DTS feature is the primary difference among the transgenic plasmids and the effect of plasmid size on association/disassociation kinetics was still apparent when comparing the smaller noDTS to the larger SMGADTS, the DTS feature of the SV40DTS plasmid appeared to drive the transfection enhancement.

When comparing the transfection performance of noDTS and SMGADTS in differentiated SH-SY5Y cells across the tested vectors, the noDTS plasmid surpassed the SMGADTS in terms of efficiency (see Figure 3A). The noDTS plasmid lacks a functional DNA targeting sequence and the SMGA plasmid contains a DTS that is only active in smooth muscle cells expressing the gamma actin cytoskeletal filament; accordingly, neither vector is theoretically capable of nuclear localization in the neuroblastoma cell. As concluded previously, the relative transfection performance among the two control plasmids is most likely due to the inferior binding kinetics and cytoplasmic diffusion rate of the large SMGADTS (7002 bp) plasmid, *vs.* the smaller noDTS plasmid (4609 bp).

#### Temporal Response of Transfection: Undifferentiated *vs.* Differentiated SH-SY5Y Cell Phenotypes

**Figure 4:**
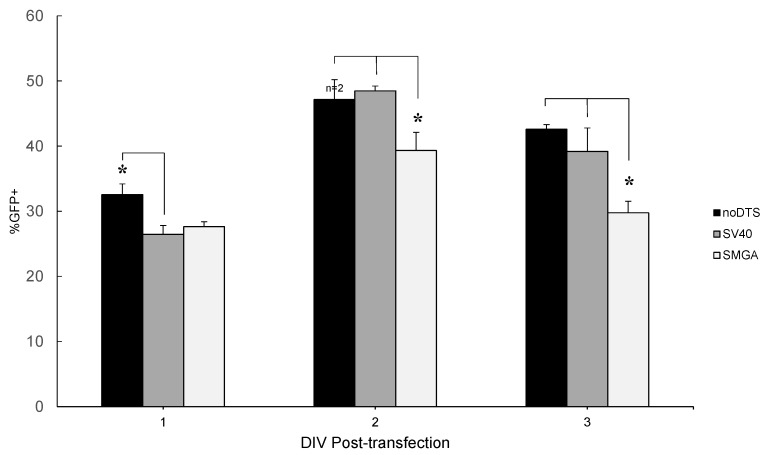
Summary graph of flow cytometry data for undifferentiated SH-SY5Y cell transfection efficiency using the Neuromag nanoparticle vector assayed at DIV 1, 2 and 3 post-transfection. Nanomagnetic plasmid delivery was conducted with the Magnefect-nano II™ controller unit with the transfection parameters of 2 Hz frequency, 0.2 mm displacement and a duration of 3600 cycles or 30 min. Error expressed as standard deviation from the mean, where *n* = 3 for all sample means except those labeled otherwise. Statistical significance of sample group means was determined by one-way ANOVA, with * *p* ≤ 0.05, where the differences in means were compared by Tukey’s Honestly Significant Data (HSD) test. There were statistically significant differences in all of the mean GFP expression values for each plasmid across the three time points sampled.

In the undifferentiated SH-SY5Y cell phenotype, the increased proliferation and metabolic rate results in a relatively rapid ascent to maximum transgene expression levels. When assayed at timepoints 24, 48 and 72 h post-transfection, the transgene expression appears to reach a maximum at 48 h as shown by the flow cytometry data presented in Figure 4. Also, the flow cytometry data reveals statistically significant attenuation in GFP expression for all three reporter plasmids when assayed at 72 h post-transfection. These observations are in accord with other researchers, who have noted that CMV driven transgene expression peaks at 24 h and begins to diminish by 48 h post-transfection for liver cells *in vivo* [30]. Furthermore, additional research studies have concluded that the mechanism of initial transgene expression attenuation after 24 h occurs primarily through promoter silencing, rather than enzymatic degradation or methylation [31,32].

**Figure 5:**
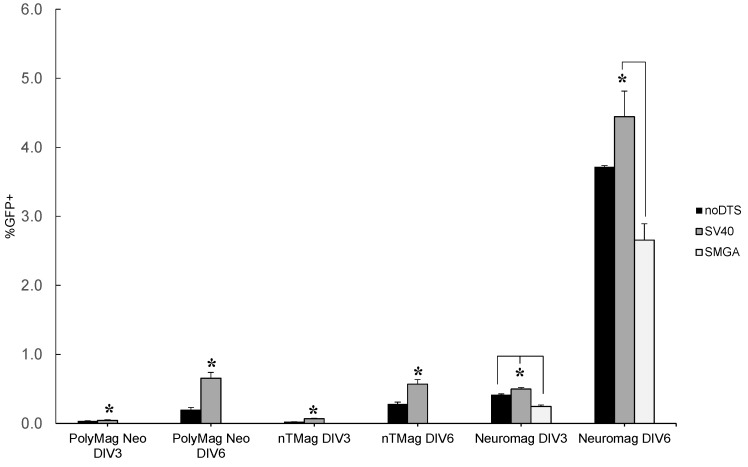
Comparison of DIV 3 and 6 flow cytometry data for differentiated SH-SY5Y cells transfected with the PolyMag Neo, nTMag and Neuromag vectors. The PolyMag Neo and nTmag nanoparticle vectors were investigated for transfection efficiency when combined with the noDTS and SV40 plasmids, but not the SMGADTS plasmid. Nanomagnetic plasmid delivery was conducted with the Magnefect-nano II™ controller unit with the transfection parameters of 2 Hz frequency, 0.2 mm displacement and a duration of 3600 cycles or 30 min. Error expressed as standard deviation from the mean, where *n* = 3 for all sample means. Statistical significance within sample groups determined using 2-sample *t*-test, with * *p* ≤ 0.05. Statistical significance of DIV 3 *vs.* DIV 6 data was determined by one-way ANOVA with * *p* ≤ 0.05, where the differences in means was compared by Tukey’s HSD test.

Transgene expression levels in the post-mitotic differentiated SH-SY5Y cell were significantly lowered and temporally delayed when compared to the undifferentiated phenotype. For all three MNP vectors tested in Figure 5, the overall GFP transgene level increased from DIV 3 to 6 post-transfection. This observation suggests that GFP transgene expression was delayed compared to identical transfection conditions in its undifferentiated precursor, where expression peaks at 48 h (see Figure 4). Furthermore, the DTS-containing SV40 plasmid outperformed the noDTS plasmid at DIV 3 and DIV 6 post-transfection for all conditions tested on differentiated neuroblastoma cells. Next, when comparing the relative performance of the two plasmids with regard to the two assaying time points, the superior transfection of the SV40DTS plasmid over controls was more pronounced for data collected at DIV 6 post-transfection. Presumably, the relative improvement in transfection efficiency was due to nuclear translocation of the SV40 plasmid, where this effect may not have fully manifested until DIV 6 post-transfection.

The Neuromag vector outperformed both nTMag and PolyMag Neo by an order of magnitude, with respect to transfection efficiency of differentiated SH-SY5Y cells. Neuromag most likely outclassed other tested vectors because this nanoparticle vector is optimized for transfection of neural cell phenotypes. Figure 5 displays a summary of the flow cytometry results for gene delivery by the nTMag, PolyMag Neo and Neuromag vectors with differentiated SH-SY5Y cells assayed at both DIV 3 and DIV 6 post-transfection. While transfection of undifferentiated neuroblastoma cells resulted in inconsistent relative performance of pDNAs tested, the superior performance of the SV40 plasmid *vs.* controls became more apparent in the differentiated SH-SY5Y cell. Furthermore, Lipofectamine™-2000 displayed obvious cytotoxicity compared to the nanoparticle vectors tested, according to the microscopy images (see Figure 3C). As shown in Figure 5, the SV40DTS outperformed the control plasmids in all vector platforms tested. Note, that the statistical comparison between noDTS and SV40DTS delivered by Neuromag and assayed at DIV 6 generated a *p*-value of 0.077. These observations suggest that the difference in performance follows the same trend as observed in Figure 5, where the SV40DTS plasmid outperformed all of the non-functional DTS control plasmids at both assay time points. The post-mitotic nature of the differentiated neuroblastoma introduces the nuclear membrane as a constant barrier to transfection; accordingly, cytoplasmic delivery of more copies of noDTS per unit vector was overshadowed by the DTS-mediated nuclear translocation of SV40DTS.

The undifferentiated SH-SY5Y cell phenotype reached a maximum expression level at 48 h and showed signs of attenuated transgene expression levels thereafter with the Neuromag vector (see Figure 4). However, the time-course of transgene expression level in the differentiated SH-SY5Y cell phenotype varies significantly from the undifferentiated precursor. Specifically, the differentiated SH-SY5Y cell phenotype appears to reach a maximum transfection level at DIV 6 post-transfection, when compared to the earlier time point of DIV 3 post-transfection. This latent transgene expression time-course may arise due to the reduced metabolic and proliferation rate of the differentiated SH-SY5Y cell phenotype compared to the mitotic undifferentiated neuroblastoma. Additionally, a relatively slower delivery of transgenic pDNA to the cell nucleus via DTS-based nuclear translocation may delay the differentiated SH-SY5Y cell phenotype from silencing the CMV promoter driving transgene reporter expression.

## 3. Experimental Section

### 3.1. SH-SY5Y Cell Culture and Differentiation

SH-SY5Y cells (ATCC^®^ CRL-2266, Manassas, VA, USA) were cultured in DMEM/HAM F12 50/50 media with l-glutamine (Cellgro, Manassas, VA, USA), supplemented with 10% FBS (Cellgro, Manassas, VA, USA) and 1% penicillin/streptomycin 100X solution (Cellgro, Manassas, VA, USA). A mammalian cell incubator (Fisher Scientific, Waltham, MA, USA) maintained culture conditions at 37 °C and 5% atmospheric CO_2_ and the ATCC CRL-2266 protocol was implemented for routine neuroblastoma subcultivation procedures [33].

SH-SY5Y differentiation induction via all-*trans* retinoic acid (ATRA) administration followed by brain-derived neurotrophic factor (BDNF) produced a dopaminergic phenotype. Briefly, induction of the “pre-differentiated” SH-SY5Y cell phenotype was accomplished by decreasing the FBS concentration to 2% with concurrent administration of 10 µM ATRA (Sigma, St. Louis, MO, USA) in a dimethyl sulfoxide (DMSO) vehicle (Sigma, St. Louis, MO, USA). SH-SY5Y culture media was replenished with 50% fresh culture media every 48 h for 6 days consisting of DMEM/F12 supplemented with 1% penicillin/streptomycin and 10 µM ATRA. This cell culture media changing regimen logarithmically decreased the FBS concentration over the course of culture differentiation to reach a final FBS concentration of 0.5% and ensure ATRA chemical viability at a constant concentration of 10 µM [34].

Upon conclusion of day 6 of ATRA treatment of SH-SY5Y cells, the pre-differentiated cells were directed toward a dopaminergic phenotype by: changing the cell culture media to remove any residual ATRA; administration of 50 ng/mL BDNF (Peprotech, Rocky Hill, NJ, USA); and maintenance in FBS at 0.5% and penicillin/streptomycin at 1% *w*/*v*. In accord with the scientific literature about differentiated SH-SY5Y cell culture, media was replenished every 3 days to ensure sufficient *in vitro* BDNF concentration for proper culture differentiation and survival. After 6 days of BDNF treatment, the dopaminergic-like SH-SY5Y cells were subcultivated into 24-well plates for transfection experiments [21].

### 3.2. SH-SY5Y Cell Characterization

SH-SY5Y cell characterization of the undifferentiated and differentiation phenotypes were confirmed through immunocytochemistry. Briefly, the adherent undifferentiated, ATRA-differentiated and BDNF-differentiated SH-SY5Y cells were cultured in 8-well glass chamber slides (Lab-Tek, Thermo Fisher Scientific, Waltham, MA, USA). After media aspiration, the chambers containing the SH-SY5Y cells were washed twice with 1X Dulbecco’s phosphate-buffered saline (DPBS, Cellgro, Manassas, VA, USA). Then, the cells were fixed in a solution of freshly prepared and filtered 4% paraformaldehyde (PFA) solution (Sigma, St. Louis, MO, USA) for 10 min at room temperature (r.t. = ~23 °C). After aspirating the PFA and performing two additional DPBS washes, the neuroblastoma cells were permeabilized with anhydrous 99.8% methanol (Sigma, St. Louis, MO, USA) for 10 min on ice (0 °C). After aspirating the permeabilization solution, the cells were incubated in the primary antibody solution overnight at 4 °C on a rotational shaker set at minimum speed. The primary antibody solution was prepared from a 1:50 dilution of both primary antibody stock solution with 1% *w*/*v* BSA (Sigma, St. Louis, MO, USA) in DPBS.

After incubation, the SH-SY5Y cells were washed twice with DPBS to remove any unbound primary antibodies. Next, the nuclei were stained for 60 s using Hoechst 33258 dye solution (2.5 µg/mL) prepared from H33258 powder (Sigma, St. Louis, MO, USA) and resuspended in distilled H_2_O. Upon conclusion of the nuclear staining step, two additional DPBS washes were performed to remove excess H33258 dye. Then, differential interference contrast (DIC) and fluorescent microscopy using the GFP, RFP and DAPI excitation and emission spectrum was performed immediately on the stained neuroblastoma cells. The primary antibodies used in this study were the Anti-beta III Tubulin (2G10) (Abcam, Cambridge, UK) directly conjugated with FITC and Anti-NeuN-PE (Millipore, Billerica, MA, USA) directly conjugated with phycoerythrin (PE). Samples were imaged using the EVOS^®^FL digital inverted fluorescence microscope using the DIC plus GFP, RFP and DAPI light cubes at 40–4000×. PFA and PFA plus methanol only treated samples were also imaged without antibody treatment as a control for background fluorescence signal level (data not shown) [35].

### 3.3. MNP Vector Characterization

#### 3.3.1. Hydrodynamic Diameter

The hydrodynamic diameter of the nTMag, PolyMag Neo and Neuromag MNP vectors were quantified using nanoparticle tracking analysis (NTA) technology. Samples of 1 mL were prepared for analysis by diluting 1 µL of PolyMag Neo, Neuromag, or 2 µL of nTMag stock solution with 999 and 998 µL, respectively in molecular biological grade DNase/RNase-free distilled H_2_O (Fisher Scientific, Waltham, MA, USA). Samples were then loaded into the fluidic chamber of the Nanosight instrument (Nanosight Technology, London, UK), placed onto the microscope stage and 60 s of video was acquired by an attached EMCCD camera instrument with sample illumination from a blue laser. The resulting video was analyzed using the Nanosight NTA 2.3 software with a particle detection threshold of 10, on a Dell Optiplex 790 running 32-bit Windows 7 Professional SP1 [36].

#### 3.3.2. Zeta Potential

The zeta potential of the nTMag, PolyMag Neo and Neuromag nanoparticle vectors were quantified with the ZetaPlus zeta potential analyzer (Brookhaven Instruments Corporation, Holtsville, NY, USA). A 1.8 mL working solution of MNPs was prepared using 20 µL PolyMag Neo or Neuromag nanoparticle stock solution and 1780 µL of dilution solution for each condition. For the more dilute nTMag nanoparticle stock solution, a working solution was prepared using 40 µL nTMag nanoparticle stock solution and 1760 µL of dilution solution. The dilution solution consisted of molecular biological grade DNase/RNase-free distilled H_2_O (Fisher Scientific, Waltham, MA, USA) adjusted to a calculated pH of 7.76 by adding 10.23 µL of 0.1 mM KOH (Sigma, St. Louis, MO, USA).

### 3.4. Magnetic Nanoparticle-Based DNA Transfection

Three plasmid reporter constructs containing green fluorescent protein (GFP) genes: pCMV-eGFP-SV40noDTS, pCMV-eGFP-SV40DTS or pCMV-eGFP-SMGA2294 were each suspended individually in 100 µL of Brainbits Transfection Medium. The plasmid DNA suspension solutions were then combined with γ-Fe_2_O_3_ nTMag (Nanotherics, Staffordshire, UK), PolyMag Neo (Oz Biosciences, San Diego, CA, USA) or Neuromag (Oz Biosciences, San Diego, CA, USA) magnetic nanoparticle vectors and then incubated for 15 min at r.t. (~23 °C). Ultimately, 7 of 9 possible nanoparticle-DNA conjugates were formed, since Polymag Neo-SMGA and nTMag-SMGA complexes were not produced for subsequent experimentation. As a positive control, Lipofectamine™-2000 (Life Technologies, Carlsbad, CA, USA) was also conjugated in parallel with the same reporter plasmids in a 20 min incubation at *ca*. 23 °C (r.t.). The mass of plasmid DNA for transfecting a single well in a 24-well cell culture plate was held constant at a value of 0.5 µg. The respective amount of vector conjugated with this amount of plasmid DNA was dependent on an empirically derived ratio (data not shown). Specifically, 1.25 µL Lipofectamine™-2000, 0.5 µL Polymag Neo, 0.5 µL nTMag and 1.7 µL Neuromag were conjugated with 0.5 µg of the noDTS, SV40DTS or SMGA plasmid. Next, complete media was used to increase conjugation reaction volumes to a working volume of 400 µL per culture plate well for transfection. Then, the wells of the 24-well culture media plate containing the SH-SY5Y cells for transfection were aspirated and replaced with 400 µL of the MNP-pDNA complex solution. Next, the culture plate was affixed to the Magnefect-nano II™ array sample holder and placed into a cell culture incubator for 30 min. Last, the oscillation program was initiated via the Magnefect-nano II™ controller unit, with the transfection parameters of 2 Hz frequency, 0.2 mm displacement and a duration of 3600 cycles or 30 min [4,7].

### 3.5. Transfection Efficiency Quantification

Approximately 24, 48 and 72 h after undifferentiated SH-SY5Y cell transfection, or 3 and 6 days following differentiated SH-SY5Y cell transfection, cells were assayed for transgene expression. Differential interface contrast (DIC) and fluorescent images of transfected SH-SY5Y cells were acquired and overlayed for at least 3 fields of view per culture plate well using the EVOS^®^FL Digital Inverted Fluorescence microscope (Life Technologies, Carlsbad, CA, USA). Finally, transfected cells were trypsinized, centrifuged at 200 RCF, washed with DPBS and resuspended in a cuvette with *ca*. 300 µL of 1% BSA (Sigma, St. Louis, MO, USA) in DPBS. Following trypsinization, all sample preparation steps described herein were performed on ice (0 °C) and then run individually through a iCyte Eclipse 488 nm-laser flow cytometer (Sony, Tokyo, Japan) to reduce cell clumping and improve measurement accuracy. Flow cytometry raw FCS file data was gated and tabulated into spreadsheets using Flowjo software version 10.0.7 (Flowjo, Ashland, OR, USA).

### 3.6. Statistical Analysis of Data

Statistical significance of the flow cytometry data was determined by either the two-sample *t*-test assuming unequal variance with a confidence interval of * *p* ≤ 0.05, or one-way ANOVA with a confidence interval of * *p* ≤ 0.05 with variance quantification by Tukey’s HSD test. The statistical tests were performed using the statistical software Minitab version 17 (Minitab Inc., State College, PA, USA).

## 4. Conclusions

The SH-SY5Y cell line serves as a model primary neuron for a variety of experimental purposes. In the present study, transfection of the two neuroblastoma phenotypes strongly suggests activity of the SV40-derived DTS feature for pDNA nuclear translocation. Next, temporal analysis of transfection outcomes in the two neuroblastoma phenotypes reveals a delayed transgene expression in post-mitotic SH-SY5Y cells. The non-specific DTS sequence appeared to enhance transfection in the differentiated, but not the undifferentiated neuroblastoma cells. While observations strongly suggest that improvement in transfection efficiency was due to the presence of a DTS sequence in the transfecting plasmid, the differences among these groups cannot be attributed solely to a plasmid-nuclear targeting sequence. Conducting future experiments designed to explore the uptake of the vector and plasmid combinations described herein, *vs.* the effect of DTS-mediated nuclear targeting would elucidate the mechanisms and their respective contribution to transfection improvement. Further investigation into the time course of gene expression efficiency in the differentiated SH-SY5Y cell may provide additional neuroblastoma transfection outcome optimization. Future studies could also aim to explore the transfection potential of neuron-specific DTS sequences, which will likely outperform the DTS-based nuclear translocation of the SV40 plasmid in neuroblastoma cells.

## Abbreviations

ATRA: all-*trans* retinoic acid; BDNF: brain-derived neurotrophic factor; DIC: differential interface contrast (microscopy); DIV: days *in vitro*; DMSO: dimethyl sulfoxide; DPBS: Dulbecco’s phosphate-buffered saline; DTS: DNA targeting sequence; GFP: green fluorescent protein; MNP: magnetic nanoparticle; NTA: nanoparticle tracking analysis; NLSs: nuclear localization signals; NPC: nuclear pore complex; PFA: paraformaldehyde; pDNA: plasmid DNA; PE: phycoerythrin; SMGA: smooth muscle gamma actin; SV40: simian vacuolating virus 40.
